# Chylous Ascites Developing Into Bilateral Chylothorax in High-Grade Lymphoma: A Case Report

**DOI:** 10.7759/cureus.37344

**Published:** 2023-04-09

**Authors:** Jonathan T Avon, Allison J Gerrard, Brijesh B Patel

**Affiliations:** 1 Pulmonary and Critical Care Medicine, Carilion Clinic, Christiansburg, USA

**Keywords:** temporal relationship, pleural effusion, non-traumatic, lymphatic fluid, thoracentesis, malignant ascites, bilateral chylothorax, non hodgkin's lymphoma, chylous ascites

## Abstract

Chylothorax and chylous ascites occur when lymphatic fluid accumulates in the pleural space or peritoneum, respectively. They are classified as either traumatic or non-traumatic, and lymphomas are the most common non-traumatic cause. Lymphomas can obstruct the lymphatic architecture causing lipid-rich chyle to leak out below the level of the obstructing mass. Bilateral chylothoraces presenting in the presence of chylous ascites, secondary to Non-Hodgkin Lymphoma, are rare. We describe a case of a 55-year-old man with recurring large-volume chylous ascites secondary to Non-Hodgkin lymphoma who developed bilateral chylothoraces. Initially, he presented with dyspnea and hypoxia and was found to have bilateral pleural effusions, requiring bilateral thoracentesis for diagnostic and therapeutic management. The fluid removed from the pleural space was found to be lymphatic fluid, and the patient was eventually discharged home with instructions to follow up with oncology for further management. The case reveals a temporal relationship where a huge volume of chylous ascites develops into a chylothorax.

## Introduction

Chylothorax is a rare condition in which triglyceride-rich lymph, known as chyle, leaks into the pleural cavity. Chyle is created by the small intestine and carried by the thoracic duct to the junction of the left subclavian and internal jugular vein [1]. There, it enters systemic circulation carrying dietary fats and proteins from the gastrointestinal tract to be used in metabolic processes [1]. Both traumatic and non-traumatic etiologies can cause chylothorax. Seventy percent of chylothoraces are non-traumatic, with lymphomas representing the most common etiology [1,2]. Chylothoraces often occur unilaterally, while bilateral effusions occur in only 16% of cases [3]. The prevalence of pleural effusions in lymphomas is 20%, with chylous effusions occurring only 12% of the time [4]. Pleural effusions may present with dyspnea, cough, and pain and may be accompanied by fever and superior vena cava syndrome [4]. Bilateral chylothoraces can develop secondary to large-volume chylous ascites in patients with lymphatic obstruction due to Non-Hodgkin Lymphoma.

Chylous ascites are the accumulation of chyle within the peritoneum caused by an interruption of normal lymphatic flow below the diaphragm. This rare phenomenon can be caused by cirrhosis, malignancy, or trauma to the lymphatic architecture [5]. Malignancy and cirrhosis account for two-thirds of all cases of chylous ascites in the Western world [5]. Patients with malignancy-induced ascites can require frequent, large-volume paracentesis. We present the case of a 55-year-old Caucasian male with large-volume chylous ascites secondary to Non-Hodgkin Lymphoma leading to bilateral chylothoraces. These complications required frequent paracentesis with the removal of up to 39.5 liters and thoracentesis with the removal of 1.5-2.7 liters.

## Case presentation

**Figure 1:**
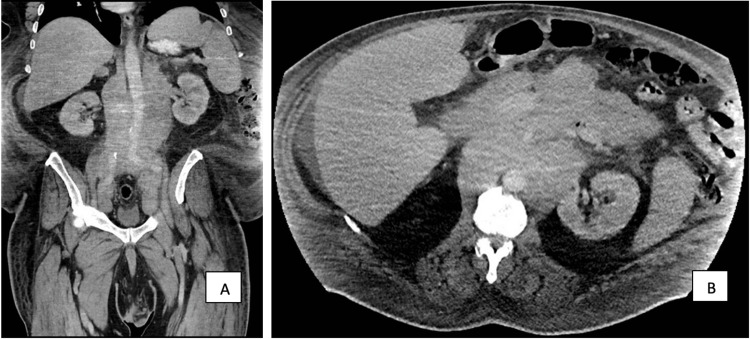
Abdominal CT showing large lymphomatous mass: (A) coronal, (B) axial.

A 55-year-old male presented to a scheduled paracentesis with worsening dyspnea and hypoxia. The patient was diagnosed four months prior with a high-grade follicular lymphoma after a large lymphomatous abdominal mass was discovered on a CT scan (Figure 1). He required weekly paracentesis, which yielded large volumes of chylous fluid, the largest being 39,500mL. His oxygen saturation was 72% on room air as he was prepped for paracentesis. His physical examination revealed bilateral decreased breath sounds and tachypnea. The patient’s oxygen saturation improved to 88% on 4L of oxygen through a nasal cannula. He was sent to the emergency department after 7,700mL of turbid white fluid was removed.

**Figure 2:**
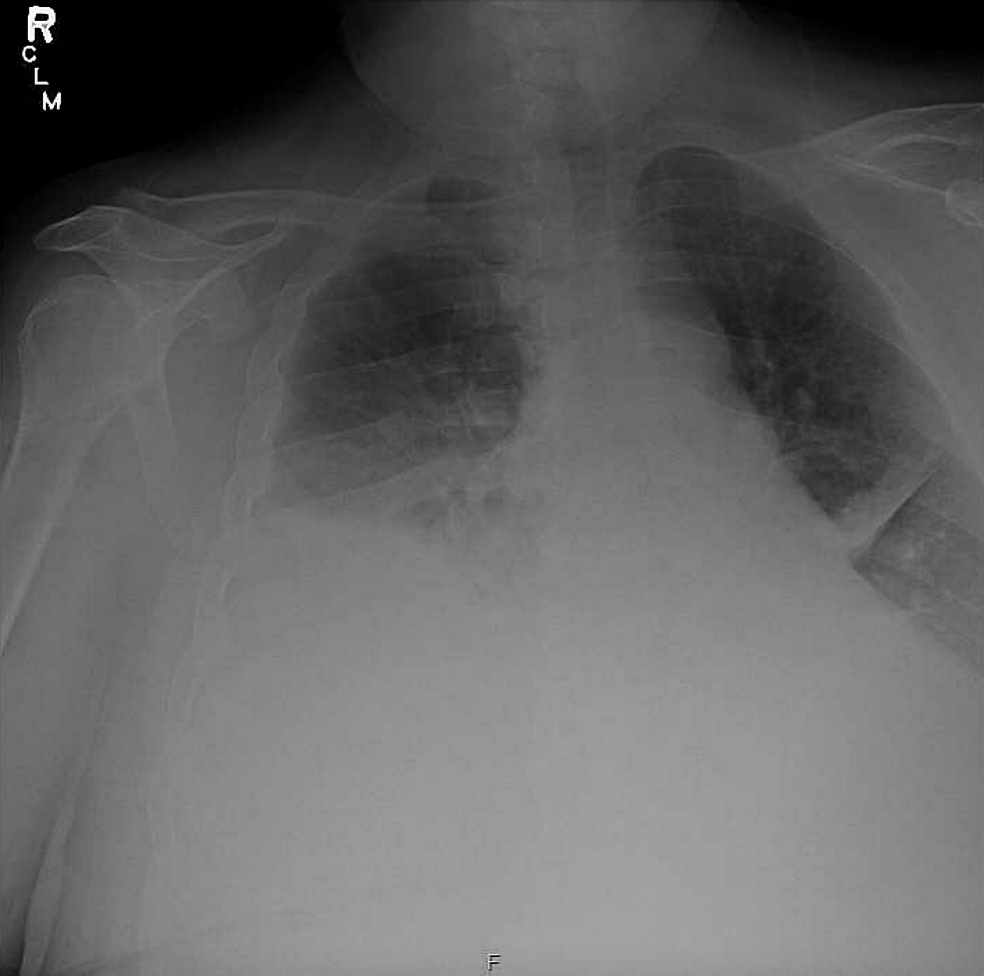
Chest radiograph showing bilateral pleural effusion

The patient mentioned that he was experiencing worsening shortness of breath and, upon physical exam, was found to have labored breathing. A chest radiograph showed an enlarged heart with moderate bilateral pleural effusions (Figure 2). A thoracentesis was then performed for diagnostic and therapeutic purposes, and 1500mL of turbid white fluid was removed from his right pleura. After the initial thoracentesis, he remained hypoxic. A subsequent CTA was completed and showed bilateral pulmonary emboli and large bilateral pleural effusions. The patient required heated high-flow oxygen and was started on 10mg of apixaban.

**Figure 3:**
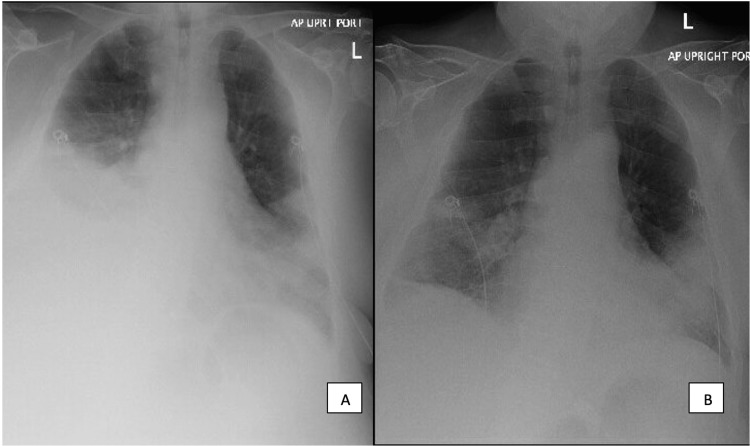
Chest radiograph after (A) left thoracentesis, (B) right thoracentesis.

The patient was admitted to the intensive care unit, where a left thoracentesis was performed, which yielded 2,700mL of lymphatic fluid. A repeat chest radiograph showed a large right pleural effusion (Figure 3a). A right-sided thoracentesis was subsequently performed and yielded 2,400mL of lymphatic fluid. The chest radiograph was repeated (Figure 3b). The patient was experiencing less dyspnea following the thoracentesis procedures. Thoracentesis lab results showed lactate dehydrogenase 190 IU/liter, total protein 2.6 g/dL, glucose 176 mg/dL, pH 7.86, and white blood cells 761, confirming the identity of the fluid as chyle. Triglyceride levels were not measured, but previous paracentesis results showed triglyceride levels greater than 2000mg/dL, further confirming the source of the fluid. The patient was discharged with home oxygen, apixaban therapy, and instructions to follow up with oncology for continued lymphoma treatment.

## Discussion

Patients with recurrent ascites can require frequent paracentesis for symptomatic improvement and quality of life. Our patient required weekly paracentesis, with large volumes of chylous ascites removed. Nearly 40 liters of chylous ascites were rarely removed from the peritoneum during a scheduled paracentesis, and the patient tolerated the procedure well. To our knowledge, a volume as large as nearly 40 liters has never been documented in the medical literature. The significant chylous ascites, including bilateral chylothoraces, led to further complications in the presented patient. 

A chylothorax typically develops from injury or obstruction to the thoracic duct within the thoracic cavity. Depending on where the thoracic duct is obstructed, chyle can accumulate in the left or right pleural space [6]. The thoracic duct begins at the L1-L2 vertebral level and travels into the posterior mediastinum after passing through the aortic hiatus in the diaphragm [5]. It continues cranially on the right side of the vertebral column until it reaches the T5 vertebrae, where it crosses over to the left superior mediastinum [5]. A right-sided chylothorax can occur if a thoracic duct obstruction occurs below T5. However, a left-sided chylothorax is more likely if the obstruction occurs superior to the T5 vertebral level [6]. In this patient, bilateral pleural effusions in the presence of chylous ascites suggest a trans-diaphragmatic mechanism instead of a thoracic duct obstruction above the diaphragm level.

In Non-Hodgkin Lymphoma, a proposed mechanism of chyle leaking into the abdominal cavity is a malignant obstruction of lymph nodes. Malignant infiltration of lymph nodes may obstruct the flow of lymph from the gut to the cisterna chili causing subserosal lymphatics on the bowel wall to become dilated and leaky [7]. It is hypothesized that fluid movement from the peritoneum to the pleural space could occur across collateral lymphatic channels that penetrate the diaphragm [8].

A more likely explanation is the presence of trans-diaphragmatic defects that allow fluid to cross into the pleural cavity. The pleural space maintains a negative intrathoracic pressure to keep the lungs inflated. Abdominal distension from ascites causes an even larger pressure gradient across the diaphragm than the existing one [9]. This gradient could cause fluid to leak through natural diaphragmatic openings, such as those around the major vessels, esophagus, or through a diaphragmatic hernia [9]. Increased abdominal pressure has also been shown to cause small portions of the peritoneum to herniate through gaps in the muscle fibers of the diaphragm [8]. These herniations can rupture, resulting in direct communication between the pleural cavity and the peritoneum [8].

## Conclusions

This unique case reveals a temporal relationship where a large volume of chylous ascites develops into a chylothorax. This rare phenomenon should be suspected in patients with large obstructive lymphomas resulting in recurring chylous ascites. Chyle in the peritoneum before the pleura suggests a trans-diaphragmatic mechanism.
